# Reproducibility of Glutamate, Glutathione, and GABA Measurements *in vivo* by Single-Voxel STEAM Magnetic Resonance Spectroscopy at 7-Tesla in Healthy Individuals

**DOI:** 10.3389/fnins.2020.566643

**Published:** 2020-09-15

**Authors:** Ofer M. Gonen, Bradford A. Moffat, Patrick Kwan, Terence J. O’Brien, Patricia M. Desmond, Elaine Lui

**Affiliations:** ^1^Department of Neurology, The Royal Melbourne Hospital, Parkville, VIC, Australia; ^2^Department of Medicine and Radiology, The University of Melbourne, Parkville, VIC, Australia; ^3^Department of Neurology, The Alfred Hospital, Melbourne, VIC, Australia; ^4^Department of Neuroscience, Central Clinical School, Monash University, Melbourne, VIC, Australia; ^5^Department of Radiology, The Royal Melbourne Hospital, Parkville, VIC, Australia

**Keywords:** magnetic resonance spectroscopy, STEAM, default mode network, posterior cingulate cortex, precuneus, reproducibility

## Abstract

**Background and Purpose:**

Derangements in brain glutamate, glutathione, and γ-amino butyric acid (GABA) are implicated in a range of neurological disorders. Reliable methods to measure these compounds non-invasively *in vivo* are needed. We evaluated the reproducibility of their measurements in brain regions involved in the default mode network using quantitative MRS at 7-Tesla in healthy individuals.

**Methods:**

Ten right-handed healthy volunteers underwent 7-Tesla MRI scans on 2 separate days, not more than 2 weeks apart. On each day two scanning sessions took place, with a re-positioning break in between. High-resolution isotropic anatomical scans were acquired prior to each scan, followed by single-voxel ^1^H-MRS using the STEAM pulse sequence on an 8 mL midline cubic voxel, positioned over the posterior cingulate and precuneus regions. Concentrations were corrected for partial-volume effects.

**Results:**

Maximal Cramér-Rao lower bounds for glutamate, glutathione, and GABA were 2.0, 8.0, and 14.0%, respectively. Mean coefficients of variation within sessions were 5.9 ± 4.8%, 9.3 ± 7.6%, and 11.5 ± 8.8%, and between sessions were 4.6 ± 4.5%, 8.3 ± 5.7%, and 9.2 ± 8.7%, respectively. The mean (±SD) Dice’s coefficient for voxel overlap was 90 ± 4% within sessions and 86 ± 7% between sessions.

**Conclusion:**

Glutamate, glutathione, and GABA can be reliably quantified using STEAM MRS at 7-Tesla from the posterior cingulate and precuneus cortices of healthy human subjects. STEAM MRS at 7-Tesla may be used to study the metabolic behavior of this important resting-state hub in various disease states.

## Introduction

Glutamate and γ-amino butyric acid (GABA) are the major excitatory and inhibitory neurotransmitters in the human brain, respectively (Kocsis and Mattson, 1996; Benarroch, 2010). Glutathione (GSH) is the most important free radical scavenging compound in the brain and is thought to be implicated in a wide range of neurological disorders, including epilepsy, multiple sclerosis, Parkinson’s disease, and motor neuron disease (Rae and Williams, 2017). All three compounds are metabolically related, as glutamate is a precursor of both GSH and GABA. Many CNS drugs exert their effects through the glutamatergic and GABAergic systems, and therapeutic interventions for increasing the concentration of GSH in the brain are investigated in recent years (e.g., dimethyl fumarate) (Kocsis and Mattson, 1996; Benarroch, 2010; Morris et al., 2014).

Interest in quantitative MRS of the human brain is increasing due to its importance in evaluating drugs that potentially affect these metabolites. However, individual quantification of glutamate, GABA, and GSH is limited by overlapping resonances from other molecules at magnetic fields up to 4-Tesla. High-field (7-Tesla) MRI offers superior signal to noise ratio and chemical shift separation which may overcome these limitations (Tkáč et al., 2001).

Several reproducibility studies on MRS of these three metabolites have been performed, evaluating metabolite concentrations and coefficients of variation (CoV) (Wijtenburg et al., 2013, 2014, 2019a,b; Lally et al., 2016; Terpstra et al., 2016; Prinsen et al., 2017). Notably, there was variation in the number of participants, number of scans, field strength, sequences used, and acquisition parameters. In addition, the size and placement of the volume of interest were also different across studies (see Table 1). Most of these studies recruited participants within a narrow age range (e.g., young adults).

**TABLE 1:** Examples of previous MRS reproducibility studies.

**No. of subjects (repeated scans)**	**Sessions**/**Day**	**No. of days**	**Acquisition methods**/**Field strength**	**Voxel placement**	**References**
4	1	2	STEAM, MEGA-PRESS-IVS/7T	ACC, DLPFC	Wijtenburg et al., 2013
10	1	2	PR-STEAM/3T	ACC, PCC	Wijtenburg et al., 2014
13	2	2	PRESS/7T	ACC	Lally et al., 2016
6	1	4	Semi-LASER/3T + 7T	PCC, cerebellar vermis	Terpstra et al., 2016
5	1	2	Semi-LASER, STEAM/7T	Occipital	Prinsen et al., 2017
10	2	1	PRESS, SPECIAL, PR-STEAM, MEGA-PRESS/7T	ACC	Wijtenburg et al., 2019a
10	1	2	STEAM/7T	ACC, PCC	Wijtenburg et al., 2019b

*ACC, Anterior cingulate cortex; DLPFC, Dorsolateral prefrontal cortex; PCC, Posterior cingulate cortex; MEGA-PRESS, Meshcher-Garwood point resolved spectroscopy; PRESS, Point resolved spectroscopy; PR-STEAM, Phase rotation stimulated echo acquisition mode; Semi-LASER, Semi-localization by adiabatic selective refocusing; SPECIAL, Spin-echo full-intensity acquired localized spectroscopy.*

In this study we sought to determine the test-retest reproducibility of quantitative single-voxel MRS of glutamate, GABA, and GSH acquired from the PCC/precuneus region at 7T using a STEAM sequence with ultrashort TE. We chose to recruit a broad age cohort, to allow application and comparison to many neurologic diseases. We also chose to scan participants twice on two different days to assess variability both within days and between different days. MRS at 7T field strength offers higher spectral resolution, but at the cost of increased specific absorption rate (SAR) (Lei et al., 2013). By using a STEAM acquisition rather than PRESS we ameliorate some of the inherent increase in the SAR.

The volume of interest was placed in the PCC/precuneus region because of its importance as a main node of the resting-state default mode network (DMN) of the brain and its implication in a wide range of neurological and psychiatric disorders (Leech and Sharp, 2014). In addition, the disproportionately high metabolic rate of this region in comparison with other cortical areas underscores the value of evaluating the reproducibility of exact MRS quantitation from this important hub (Buckner et al., 2008).

We hypothesized that quantitative MRS of these metabolites at 7T will have high reproducibility. There is some evidence that glutamate and GABA in the PCC/precuneus are related to DMN activity. For example, a study of 24 health volunteers at 3T showed that GABA concentration in the PCC/precuneus positively correlated with task-related DMN deactivation, whereas glutamate negatively correlated with it (Hu et al., 2013). Therefore, the ability to measure the concentration of these metabolites reliably within the PCC/precuneus may assist with studying them as potential non-invasive biomarkers for medical conditions affecting the DMN (e.g., epilepsy, Alzheimer’s disease), perhaps even during a subclinical phase, and for assessment of treatment response.

## Materials and Methods

### Participants

Ten healthy, right-handed, volunteers were recruited. The participant cohort consisted of six women and four men, with a mean ± standard deviation (SD) age of 40 ± 14 years. No participant had any history of neurological or psychiatric disease. The study was approved by the Institutional Review Board (IRB) of The University of Melbourne and all participants provided written informed consent.

### Imaging Protocol

MRI scans were acquired using a 7T research scanner (Siemens Healthcare, Erlangen, Germany) with a 32-channel head-coil (Nova Medical Inc., Wilmington MA, United States). To assist with localization MP2RAGE was performed prior to each session at 0.9 mm resolution, *TR* = 4900 ms, *TE* = 2.9 ms, TI1 = 700 ms, FA1 = 5°, TI2 = 2700 ms, FA2 = 6°, acquisition time (TA) 5:54 min. After manual shimming, single-voxel 1H MRS was acquired using the STEAM method with a 20 × 20 × 20 mm cubic voxel (volume of 8 mL), *TR* = 8500 ms (except for the scans of participant 5 on day 1, for which *TR* = 9300 ms was used due to reaching a SAR limit), and *TE* = 6 ms using outer volume suppression. Thirty-two averages were used for the water-suppressed sequence (*TA* = 4:49 min) and four averages for the unsuppressed sequence (*TA* = 51 s).

We used high bandwidth pulses for the STEAM acquisition in conjunction with outer volume suppression to minimize the chemical shift artifact. In addition, we included terms fitting the remaining lipid and macromolecular contaminations in accordance with methods published by Hurd (2011).

**FIGURE 1:**
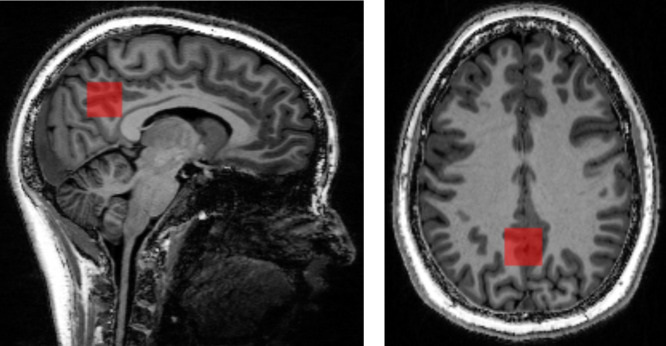
T_1_-weighted MRI with superimposed MRS voxel. left – sagittal; right – axial).

The voxel was placed in the midline, with its anterior inferior border just above the corpus callosum, its anterior superior border just dorsal to the marginal branch of the cingulate sulcus, and its posterior border ventral to the parieto-occipital sulcus, in a similar manner to the study of Kantarci et al. (2007; Figure 1).

Following several minutes’ break, during which time the patients were encouraged to move their head and reposition themselves inside the scanner, 1 mm isotropic MP2RAGE was acquired (*TA* = 4:26 min) for voxel placement using the same landmarks. Identical parameters were used for the second intra-session MRS. The above was repeated for each participant not more than 14 days after the first scanning session.

### Image Post-processing and Quantitative MRS Calculation

Metabolite concentrations were quantified using LCModel (version 6.3-0B) with a 7T basis set (Provencher, 1993). Eddy-current correction was used, and the metabolite concentrations were scaled to unsuppressed water. Cramér–Rao lower bounds (CRLB), estimated standard deviations, expressed in percent of the estimated concentrations, were calculated by LCModel.

For partial volume effect correction, binary masks were created for each MRS voxel acquired using a MATLAB script created by Mr. Bartosz Kossowski from The Polish Academy of Sciences^1^ – see acknowledgments. MATLAB version R2017b was used (MathWorks, Natick, MA, United States). The dimensions of the masks were transformed via the ANTs toolbox (version 2.3.1)^2^ to be consistent with MP2RAGE coordinates (Avants et al., 2011). The MP2RAGE anatomical images were skull-stripped and the brain extracted using FMRIB’s Brain Extraction Tool (Smith, 2002).

Each brain was then segmented using FMRIB’s Automated Segmentation Tool (FAST) into partial volume maps for gray matter, white matter, and CSF (Zhang et al., 2001). The fractions of each of these partial volume maps within each MRS voxel were determined using the fslstats utility of the FSL suite (version 5.0.10) (Jenkinson et al., 2012). The NMR-visible water concentration (mM) in the voxel was estimated by (43300f_GM_+35880f_WM_+55556f_CSF_)/(1-f_CSF_), where f_GM_, f_WM_, and f_CSF_ are the volume fractions of gray matter, white matter, and CSF in the voxel, respectively (Lee et al., 2013).

Concentrations of metabolites were reported both as corrected water-scaled values in mM units based on the above formula and as dimensionless values based on the raw LCModel output relative to total creatine (creatine + phosphocreatine) to enable comparison to previous studies.

### Reproducibility Assessment and Statistical Analysis

Intra- and inter-session reproducibility of MRS concentrations was assessed via CoV and Intraclass Correlation Coefficients (ICC) with two-way random effects for average measures (absolute agreement) (Shrout and Fleiss, 1979). For evaluation of reproducibility according to ICCs, ICC <0.40 was considered poor; between 0.40 and <0.60 fair; between 0.60 and <0.75 good; and between 0.75 and 1.00 excellent (Cicchetti, 1994). Reproducibility of the CSF volume fraction within the MRS voxel was also assessed with CoV. The Dice similarity coefficient was used to calculate the spatial overlap between the MRS voxels in different scans using FMRIB’s Brain Intensity AbNormallity Classification Algorithm (BIANCA) (Griffanti et al., 2016). Statistical analyses were conducted with Stata 15.0 (StataCorp, College Station, TX, United States).

## Results

**FIGURE 2:**
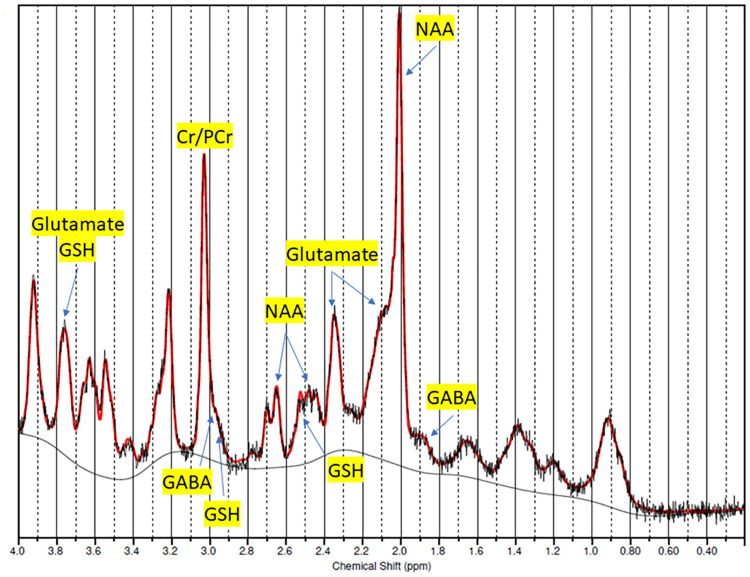
Example of a spectrum.

All participants completed the two scans separated by 4–14 days (mean ± SD was 8.9 ± 3.1 days). Mean ± SD CRLB for glutamate, GSH, and GABA were 2.0 ± 0%, 5.0 ± 0.8%, and 8.3 ± 1.6%, respectively; maximal CRLB were 2, 8, and 14%, respectively. The mean ± SD FWHM of the 40 spectra was 0.033 ± 0.004 ppm (9.8 ± 1.2 Hz), which reflects good quality acquisition (Kreis, 2004). The chemical shift artifact was 1.7 mm per ppm and mean ± SD data shift across all 40 scans was 0.026 ± 0.005 ppm. An example of one of the spectra appears in Figure 2.

**FIGURE 3:**
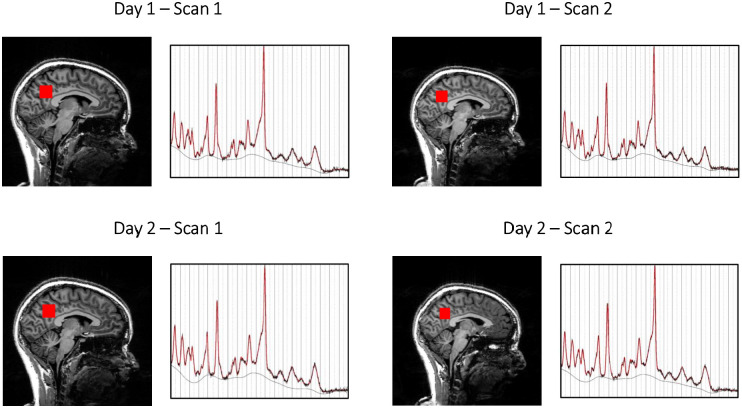
Voxel prescriptions and spectra of a single subject (subject number 10).

Mean ± SD concentrations of glutamate, GSH, and GABA scaled to unsuppressed water were 10.18 ± 1.00 mM, 1.83 ± 0.27 mM, and 1.56 ± 0.27 mM, respectively; concentrations scaled to total creatine were 1.16 ± 0.07, 0.21 ± 0.02, and 0.18 ± 0.03, respectively (Table 2). The intra and inter-session coefficients of variation for water-scaled metabolites ranged between 2.79 and 6.41% for glutamate, 6.66 and 10.82% for GSH, and 10.97 and 11.69% for GABA. ICC values were 0.32–0.75, 0.20–0.72, and 0.49–0.82, respectively (Table 3). Our results are compared to similar creatine-scaled studies in Table 4. An analysis of MRS voxel placement reproducibility between scans is included in Table 5 and an example of the four scans of a single subject (subject 10) appears in Figure 3. Concentrations of several other metabolites that were consistently acquired with good quality (CRLB < 20%) in all scans and raw uncorrected water-scaled data for all the metabolites discussed in this manuscript appear in Supplementary Tables 1, 2.

**TABLE 2:** Metabolite concentrations.

**A – Mean concentrations scaled to unsuppressed water (standard deviation)**					
**Metabolite (mM)**	**Day 1/Scan 1**	**Day 1/Scan 2**	**Day 2/Scan 1**	**Day 2/Scan 2**	**Overall**
Glutamate	10.34 (0.93)	9.69 (0.95)	10.24 (0.66)	10.45 (1.30)	10.18 (1.00)
GSH	1.86 (0.27)	1.83 (0.25)	1.80 (0.21)	1.84 (0.37)	1.83 (0.27)
GABA	1.52 (0.20)	1.48 (0.31)	1.57 (0.31)	1.65 (0.26)	1.56 (0.27)

**B – Mean concentrations scaled to creatine (standard deviation)**					
**Metabolite/Cr**	**Day 1/Scan 1**	**Day 1/Scan 2**	**Day 2/Scan 1**	**Day 2/Scan 2**	**Overall**

Glutamate	1.16 (0.07)	1.13 (0.06)	1.16 (0.07)	1.17 (0.09)	1.16 (0.07)
GSH	0.21 (0.02)	0.22 (0.03)	0.20 (0.03)	0.20 (0.02)	0.21 (0.02)
GABA	0.17 (0.02)	0.17 (0.03)	0.18 (0.03)	0.18 (0.02)	0.18 (0.03)

**TABLE 3:** Reproducibility measures.

**Type of Comparison**	**Coefficient**	**Relative to unsuppressed water**			**Relative to Creatine**		
		**Glutamate**	**GSH**	**GABA**	**Glutamate**	**GSH**	**GABA**
Within Day 1	ICC	0.72	0.66	0.49	0.44	0.33	–0.27
(Scan 1 vs. Scan 2)	CoV (%)	5.91%	7.81%	11.52%	3.56%	8.99%	13.11%
Within Day 2	ICC	0.32	0.20	0.52	0.90	0.59	0.72
(Scan 1 vs. Scan 2)	CoV (%)	5.83%	10.69%	11.42%	2.22%	6.67%	8.32%
Between-session	ICC	0.87	0.72	0.82	0.85	0.67	0.69
(Scan 1)	CoV (%)	2.79%	6.66%	7.35%	2.82%	5.69%	8.89%
Between-session	ICC	0.68	0.69	0.65	0.51	0.13	–0.35
(Scan 2)	CoV (%)	6.40%	9.84%	10.97%	4.00%	10.13%	9.91%
Overall (4 scans)	ICC	0.75	0.68	0.77	0.86	0.59	0.66
	CoV (%)	6.41%	10.82%	11.69%	3.00%	8.38%	8.06%

**TABLE 4:** Comparison to other creatine-scaled MRS studies of the PCC (Lally et al., 2016; Wijtenburg et al., 2019b).

**Type of Comparison**	**Lally et al.**			**Wijtenburg et al.**			**Gonen et al.**		
	**Glutamate**	**GSH**	**GABA**	**Glutamate**	**GSH**	**GABA**	**Glutamate**	**GSH**	**GABA**
Mean concentration day 1	1.37	0.25	0.20	1.25	0.24	0.34	1.15	0.22	0.17
CoV day 1	6.00%	14.95%	36.89%	N/A	N/A	N/A	3.56%	8.99%	13.11%
ICC (mean) day1	0.88	0.49	–0.17	N/A	N/A	N/A	0.44	0.33	–0.27
Mean concentration day 2	1.33	0.24	0.19	1.16	0.23	0.36	1.17	0.20	0.18
CoV day 2	4.77%	8.54%	29.19%	N/A	N/A	N/A	2.22%	6.67%	8.32%
ICC (mean) day2	0.94	0.88	0.37	N/A	N/A	N/A	0.90	0.59	0.72
Mean concentration 1st scans	1.38	0.25	0.20	N/A	N/A	N/A	1.16	0.21	0.18
CoV both 1st scans	6.48%	11.45%	30.92%	6.20%	14.30%	18.40%	2.82%	5.69%	8.89%
ICC (mean) both 1st scans	0.86	0.65	–0.26	N/A	N/A	N/A	0.85	0.67	0.69
Mean concentration 2nd scans	1.38	0.25	0.18	N/A	N/A	N/A	1.15	0.21	0.18
CoV both 2nd scans	7.95%	8.25%	15.43%	N/A	N/A	N/A	4.00%	10.13%	9.91%
ICC (mean) both 2nd scans	0.68	0.76	0.21	N/A	N/A	N/A	0.51	0.13	–0.35

**TABLE 5:** MRS voxel composition and overlap of individual subjects.

**Subject**	**CSF coefficient of variation**				**Dice coefficient (voxel overlap)**			
	**Day 1 (%)**	**Day 2 (%)**	**1st scans (%)**	**2nd scans (%)**	**Day 1 (%)**	**Day 2 (%)**	**1st scans (%)**	**2nd scans (%)**
1	12.3	19.2	8.8	1.8	94.5	93.8	88.8	89.6
2	14.0	12.8	9.5	8.3	90.8	90.6	79.0	76.9
3	20.2	28.0	1.1	7.0	83.9	92.2	86.5	95.6
4	9.9	7.4	0.7	18.0	92.2	93.1	77.5	72.0
5	11.2	36.7	11.0	56.5	85.7	86.2	83.5	75.8
6	14.9	47.1	2.0	61.6	85.7	89.4	87.9	88.3
7	55.6	27.3	18.0	12.9	89.5	87.0	91.5	93.1
8	2.4	2.1	8.2	3.8	83.9	95.2	89.0	96.0
9	34.1	28.8	10.3	4.8	92.2	81.8	86.3	77.3
10	66.9	11.4	27.7	53.3	93.9	92.0	91.0	94.2
Mean	24.1	22.1	9.7	22.8	89.2	90.1	86.1	85.9

## Discussion

Our study demonstrated good reproducibility of glutamate, GSH, and GABA concentrations measured from the PCC/precuneus at 7T using STEAM, as measured by CoV in a population of subjects with a broader age range in comparison with other studies. ICC results varied, ranging between poor and excellent across different comparisons, with the lowest values recorded between the sessions of the 2nd day, suggesting a substantial effect of the random repositioning during that day. However, the overall ICC values, and in particular the ICCs of the 1^st^ scans after standard positioning in the scanner on both days ranged, for the most part, between good and excellent, depending on the metabolite and scaling.

Within- and between-sessions reproducibility measures were not explored in most other 7T studies. A notable exception is the study of Lally et al. (2016) who measured creatine-scaled metabolite ratios in the ACC twice on two different days in 26 subjects ranging in age from 20 to 54 years using PRESS. Other exceptions are the studies of Stephenson et al. (2011) and Cai et al. (2012) who studied intra-session and inter-session reproducibility of MRS quantitation of glutamate and GABA, without GSH quantification. Our study, therefore, is novel as it is a 7T quantitative STEAM study with a 2^∗^2 design, assessing reproducibility by CoV and ICC of all three J-coupled metabolites (glutamate, GSH, and GABA) in the PCC/precuneus.

Prinsen et al. (2017) who acquired MRS at 7T from the midline occipital cortex, reported CoV of 3.2, 7.8, and 9.5%, for glutamate, GSH, and GABA, respectively. Their cohort consisted of five participants with a mean age of 32 years (range 24–40) who were scanned once only on two different days, with mixed sequences (STEAM for glutamate; Semi-Localized by Adiabatic Selective Refocusing for GSH and GABA) (Prinsen et al., 2017). Terpstra et al. (2016) did not report exact CoV figures for their 7T PCC study, but judging by their bar charts, our CoV values seem to be superior.

Wijtenburg et al. (2013; 2014; 2019a; 2019b) studied the reproducibility of brain MRS in a series of publications at 3T and at 7T in groups of volunteers who were scanned twice using several different sequences. Metabolites acquired via STEAM from the PCC at 7T were from an older cohort scanned twice 2–3 months apart using a voxel of 1.6 × 2.0 × 2.8 cm and were reported as ratios relative to total creatine (creatine + phosphocreatine) without exact quantification in water-scaled values (Wijtenburg et al., 2019b). No ICC values were reported for this cohort.

In our study we reported ratios of metabolite concentrations relative to total creatine, to enable comparison with other similar studies. However, the percentages of gray matter, white matter, and CSF may vary in the volume of interest as a function of voxel placement and patient characteristics, and the concentration of creatine itself can vary with age (Suri et al., 2017). Therefore, we also calculated exact concentrations of metabolites scaled to water with partial volume correction according to segmentation to gray matter, white matter, and CSF, as outlined above, which renders the results more useful for future quantitative MRS biomarker studies. This also allows comparison to other studies that evaluated water-scaled metabolite concentrations (Wijtenburg et al., 2014, 2019a; Terpstra et al., 2016; Prinsen et al., 2017).

We chose STEAM, which has the advantage of a lower SAR compared to PRESS because of using 90° rather than 180° excitation pulses, at the expense of a lower signal-to-noise ratio (SNR) (Öz et al., 2020). A lower SAR is beneficial as it enables the inclusion of other sequences as part of the MRI session. As the SNR improves at 7T, STEAM becomes more advantageous (Lei et al., 2013). Several new MRS sequences were introduced in recent years such as the spin-echo full-intensity acquired localized (SPECIAL) and the localization by adiabatic selective refocusing (LASER) (Lin et al., 2014; Mekle et al., 2009). However, while these sequences and their semi-adiabatic versions have superior localization performance, they also have limitations. The semi-adiabatic LASER sequence is most suitable when TE values of 25–30 ms are acceptable, whereas the semi-adiabatic SPECIAL sequence is suitable for shorter TE values but at the expense of increased susceptibility to subject motion (Öz et al., 2020). Therefore, another advantage of STEAM with ultrashort TE, as used in our study, is its applicability in cases of subjects prone to move during the scan (Öz et al., 2020). This provides further support to the importance of studying MRS reproducibility of STEAM with ultrashort TE, as the results can be applied in studies of patients with conditions such as epilepsy and various forms of dementia, in which subject motion is expected. Our results are, therefore, encouraging in the sense that despite intrinsically lower signal to noise ratio, the reproducibility of STEAM at 7T with ultrashort TE is not inferior.

Our metabolite ratios, when scaled to total creatine, were somewhat lower in comparison with Lally et al’s values (Table 4; Lally et al., 2016). Glutamate was 0.22 and 0.16 units lower on the first and second days, respectively (16.1 and 12.0% lower, respectively). Similarly, GSH was 0.03 and 0.04 units lower (12.0 and 16.7%, respectively), and GABA was 0.03 and 0.01 units lower (15.0 and 5.3%, respectively).

Although Lally’s study was also of healthy subjects at 7T, this is not surprising, with the intrinsic lower SNR of a STEAM MRS sequence compared to the PRESS. It is also possible to at least be partially related to the difference in location of voxel placement, ours in the PCC/precuneus compared to Lally et al’s pregenual cingulate. Notably, the relative contribution of creatine and phosphocreatine to the spectral peaks used for metabolite quantification in the study of Lally et al. (2016) is not entirely clear, and this may affect the ability for direct comparison between the studies. Regardless, this is not at the expense of poorer reproducibility.

Compared to Lally’s study, our CoV are lower, for all three metabolites, and for all intra and inter-sessions, except for inter-session GSH of 2nd scans, which was 10.1% in our study and 8.3% in Lally’s. For example, our GABA intra-session CoV are 13.1 and 8.3% compared to Lally’s 36.9 and 29.2%, respectively. Similarly, our inter-session 1st scan glutamate CoV are 2.8% vs. Lally’s 6.5%, and our GSH CoV is 5.7% vs. Lally’s 11.5%.

Compared to Wijtenburg et al. (2019b)’s 7T STEAM study, our metabolite concentrations were also lower for the most part. Glutamate was 0.10 units lower on the first day and 0.01 units higher on the second day, respectively (8.4 and 0.4%, respectively). GSH was 0.02 and 0.03 units lower, respectively (10.4 and 13.0%, respectively). Our GABA concentrations were substantially lower – 0.17 and 0.18 units, respectively (50.0% for both). But still, the CoV were bigger in Wijtenburg’ study, and their mean values for creatine-scaled metabolites in the PCC region (1.6 × 2.0 × 2.8 cm voxel) were 6.2, 14.3, and 18.4% for glutamate, GSH, and GABA, respectively.

It may be the shorter TE of our STEAM sequence compared to Wijtenburg et al.’s (2019b) 7T study (6 ms compared to 14 ms) that explains our superior reproducibility, reducing signal loss due to T_2_ relaxation. Notably, we used outer volume suppression to enable shorter echo times (Chen et al., 1997). We also used longer TR (8500 ms compared to 3000 ms) which diminished signal loss due to T_1_-weighting. Finally, our improved reproducibility may also be related to our shorter interval between scan sessions (8.9 days vs. 2.4 months).

A direct comparison of water-scaled metabolite concentrations to Wijtenburg’s 2014 phase rotation STEAM study is not possible, as values in the latter were reported in “institutional units,” but we note that their CoV for glutamate, GSH and GABA between their two scans were 7.2, 8.6, and 10.5%, respectively, which were higher than values between the 1st sessions of each day in our study, but lower than our results for the 2nd sessions of each day, except for glutamate. Their ICCs for these metabolites were 0.59, 0.51, and 0.35, respectively, and in comparison, our ICCs were lower both for the intersession comparison of the 1st scans of each day, and the comparison of the 2nd scans of each day. When carefully examining the raw data of Prinsen et al. (2017) who used a 2 × 2 × 2 cm cubic voxel positioned in the mid-occipital region but largely consisting of precuneus tissue, their mean concentrations for glutamate, GSH and GABA using STEAM were 10.56 ± 0.48, 1.34 ± 0.13, and 1.38 ± 0.26 mM, comparable to our results.

The CoV in our study are not clearly lower when the metabolic concentrations are scaled to water vs. creatine. The CoV of glutamate concentration is consistently the lowest for both intra and inter-session reproducibility comparison. Both GABA and GSH have generally larger CoV than glutamate. However, GABA and GSH have much lower concentrations than glutamate. The mean (±SD) ratio of glutamate to GABA across all days was 6.60 (±0.17) and for glutamate to GSH was 5.58 (±0.33). In addition, glutamate has the lowest CRLB values. Therefore, the lower CoV of glutamate was expected.

Although some of the intra-session CoV are higher than between sessions, this is not a consistent finding across the three metabolites. This is most evident for GABA, with both intra-session CoV higher than both inter-session CoV, but when scaled to water, the absolute difference is small (i.e., highest mean intra-session CoV of 11.5% compared to highest mean inter-session CoV of 11.0%). GSH also has a mean intra-session CoV higher than its highest inter-session CoV. Again, the absolute difference is small (intra-session CoV 10.7 vs. 9.8% inter-session).

Variation in voxel placement and composition is an important contributing factor to the variation in CoV, particularly within, but also between, sessions. Three subjects had their lowest Dice coefficient and four subjects had their highest variation in CSF composition within the first day’s session, with one subject having a 55.6% variation in CSF composition in the first day’s session. Four subjects had the lowest Dice coefficients between the 2nd scans of each session. Not only were these of the lowest values (Dice between 72 and 77%), but two subjects also had the highest CSF composition variation (of up to 61.6%) between the 2nd scans of each session. Although meticulous adherence to the multiple landmarks was employed and included in our voxel positioning, our methodology also included intersession repositioning of the patient’s head in a way that accentuates interscan rotational differences. Notably, while we positioned the patients facing up for the first scan of each session, we deliberately asked them to rotate their heads during the break within sessions so that they were positioned in random angles during the second scan of each day. However, even despite this, our intrasession CoV remain smaller than Lally’s, and our Dice coefficients also remain similar to limited available literature.

Bai et al. (2017) measuring the reproducibility of voxel placement for GABA-edited MRS on 13 healthy volunteers using 9 mL cubic voxels placed over the right sensorimotor and midline occipital cortices has within-subject Dice coefficients of 86 ± 5% and 87 ± 5%, respectively, compared to our values of 89 ± 4% and 90 ± 4% within the 1st and 2nd days, respectively, and 86 ± 5% and 86 ± 9% within the first and second sessions of each day, respectively.

A limitation of this study is the MP2RAGE resolution was higher in the first scans of each day (0.9 mm isotropic) that the second scans (1 mm isotropic). This was done for reasons related to SAR, as MRS was acquired as part of a broader protocol. Nevertheless, the spectra were all acquired with an identical voxel size, and for the purposes of comparison of voxel placement, all images of each patient were resliced and co-registered to the first image of the first scan. Another point to consider is that metabolite concentrations may fluctuate according to diurnal variation, with our participants scanned at different times of the day for reasons related to their convenience (Arm et al., 2019).

In conclusion, glutamate, GSH and GABA can be reliably quantified at 7T using STEAM MRS with ultrashort TE from the posterior cingulate and precuneus cortices of healthy individuals ranging in age from young adults to elderly. This method strikes a balance between the signal to noise ratio and susceptibility to subject motion, which can occur with advanced age and in certain patient groups. Moreover, the broad age range used in our study serves as a proof of concept for acquiring MRS biomarkers in conditions affecting different age groups (e.g., schizophrenia, epilepsy, Alzheimer’s disease), focusing on the metabolic behavior of this important resting-state hub in various disease states.

## Data Availability Statement

The datasets for this article are not publicly available because the raw imaging files are stored on a secure server, as per the ethics approval. However, the numerical output of the LCModel computer program for all participants is available upon request. Requests to access the datasets should be directed to Ofer M. Gonen, Ofer.Gonen@mh.org.au.

## Ethics Statement

The studies involving human participants were reviewed and approved by the University of Melbourne Institutional Review Board. The patients/participants provided their written informed consent to participate in this study.

## Author Contributions

OG: conceptualization, methodology, investigation, formal analysis, and writing original draft. TO’B and PK: conceptualization, funding acquisition, methodology, supervision, and review of manuscript. PD and EL: conceptualization, methodology, supervision, and review of manuscript. BM: investigation, resources, formal analysis, methodology, and review of manuscript. All authors contributed to the article and approved the submitted version.

## Conflict of Interest

The authors declare that the research was conducted in the absence of any commercial or financial relationships that could be construed as a potential conflict of interest.

## Footnotes

1 https://www.nitrc.org/projects/rda2nifti/

2 http://stnava.github.io/ANTs/

## Supplementary Material

TABLE S1 Other consistently acquired metabolites. NAA, N-acetylaspartate; GLN, Glutamine; INS, Inositol; Total Cr, Creatine + Phosphocreatine.

TABLE S2 Raw uncorrected water-scaled data. GABA, γ-amino butyric acid; GLUT, Glutamate; GSH, Glutathione; NAA, N-acetylaspartate; GLN, Glutamine; INS, Inositol; Total Cr, Creatine + Phosphocreatine. For creatine-scaling divide by total creatine data columns.

## References

[B1] Arm J. Al-iedani O. Lea R. Lechner-Scott J. Ramadan S. (2019). Diurnal variability of cerebral metabolites in healthy human brain with 2D localized correlation spectroscopy (2D L-COSY). *J. Magn. Reson. Imaging* 50 592–601. 10.1002/jmri.26642 30629765

[B2] Avants B. B. Tustison N. J. Song G. Cook P. A. Klein A. Gee J. C. (2011). A reproducible evaluation of ANTs similarity metric performance in brain image registration. *Neuroimage* 54 2033–2044. 10.1016/j.neuroimage.2010.09.025 20851191 PMC3065962

[B3] Bai X. Harris A. D. Gong T. Puts N. A. J. Wang G. Schär M. (2017). Voxel placement precision for GABA-Edited magnetic resonance spectroscopy. *Open J. Radiol.* 7 35–44. 10.4236/ojrad.2017.71004 28690923 PMC5497851

[B4] Benarroch E. E. (2010). Glutamate transporters: diversity, function, and involvement in neurologic disease. *Neurology* 74 259–264. 10.1212/WNL.0b013e3181cc89e3 20083803

[B5] Buckner R. L. Andrews-Hanna J. R. Schacter D. L. (2008). The brain’s default network: anatomy, function, and relevance to disease. *Ann. N. Y. Acad. Sci.* 1124 1–38. 10.1196/annals.1440.011 18400922

[B6] Cai K. Nanga R. P. R. Lamprou L. Schinstine C. Elliott M. Hariharan H. (2012). The impact of gabapentin administration on brain gaba and glutamate concentrations: a 7T 1H-MRS study. *Neuropsychopharmacology* 37 2764–2771. 10.1038/npp.2012.142 22871916 PMC3499716

[B7] Chen Y. J. Rachamadugu S. Fernandez E. J. (1997). Three dimensional outer volume suppression for short echo time in vivo 1H spectroscopic imaging in rat brain. *Magn. Reson. Imaging* 15 839–845. 10.1016/S0730-725X(97)00044-1 9309614

[B8] Cicchetti D. V. (1994). Guidelines, criteria, and rules of thumb for evaluating normed and standardized assessment instruments in psychology. *Psychol. Assess.* 6 284–290. 10.1037//1040-3590.6.4.284

[B9] Griffanti L. Zamboni G. Khan A. Li L. Bonifacio G. Sundaresan V. (2016). BIANCA (Brain Intensity AbNormality Classification Algorithm): a new tool for automated segmentation of white matter hyperintensities. *Neuroimage* 1 191–205. 10.1016/j.neuroimage.2016.07.018 27402600 PMC5035138

[B10] Hu Y. Chen X. Gu H. Yang Y. (2013). Resting-state glutamate and GABA concentrations predict task-induced deactivation in the default mode network. *J. Neurosci.* 33 18566–18573. 10.1523/JNEUROSCI.1973-13.2013 24259578 PMC3834059

[B11] Hurd R. E. (2011). Artifacts and pitfalls in MR spectroscopy. *Clinical MR Neuroimaging: Physiological and Functional Techniques.* Cambridge: Cambridge University Press.

[B12] Jenkinson M. Beckmann C. F. Behrens T. E. J. Woolrich M. W. Smith S. M. (2012). FSL - Review. *Neuroimage* 62 782–790. 10.1016/j.neuroimage.2011.09.015 21979382

[B13] Kantarci K. Weigand S. D. Petersen R. C. Boeve B. F. Knopman D. S. Gunter J. (2007). Longitudinal 1H MRS changes in mild cognitive impairment and Alzheimer’s disease. *Neurobiol. Aging* 28 1330–1339. 10.1016/j.neurobiolaging.2006.06.018 16860440 PMC2766807

[B14] Kocsis J. D. Mattson R. H. (1996). GABA levels in the brain: a target for new antiepileptic drugs. *Neuroscientist* 2 326–334. 10.1177/107385849600200610

[B15] Kreis R. (2004). Issues of spectral quality in clinical 1H-magnetic resonance spectroscopy and a gallery of artifacts. *NMR Biomed.* 17 361–381. 10.1002/nbm.891 15468083

[B16] Lally N. An L. Banerjee D. Niciu M. J. Luckenbaugh D. A. Richards E. M. (2016). Reliability of 7T 1H-MRS measured human prefrontal cortex glutamate, glutamine, and glutathione signals using an adapted echo time optimized PRESS sequence: a between- and within-sessions investigation. *J. Magn. Reson. Imaging* 43 88–98. 10.1002/jmri.24970 26059603 PMC4671833

[B17] Lee H. Caparelli E. Li H. Mandal A. Smith S. D. Zhang S. (2013). Computerized MRS voxel registration and partial volume effects in single voxel 1H-MRS. *Magn. Reson. Imaging* 31 1197–1205. 10.1016/j.mri.2013.04.001 23659770

[B18] Leech R. Sharp D. J. (2014). The role of the posterior cingulate cortex in cognition and disease. *Brain* 137 12–32. 10.1093/brain/awt162 23869106 PMC3891440

[B19] Lei H. Xin L. Gruetter R. Mlynárik V. (2013). “Localized single-voxel magnetic resonance spectroscopy, water suppression, and novel approaches for ultrashort echo-time measurements,” in *Magnetic Resonance Spectroscopy: Tools for Neuroscience Research and Emerging Clinical Applications*, eds Stagg C. Rothman D. L. (Cambridge: Academic Press).

[B20] Lin M. Kumar A. Yang S. (2014). Two-dimensional J-resolved LASER and semi-LASER spectroscopy of human brain. *Magn. Reson. Med.* 71 911–920. 10.1002/mrm.24732 23605818 PMC4082480

[B21] Mekle R. Mlynárik V. Gambarota G. Hergt M. Krueger G. Gruetter R. (2009). MR spectroscopy of the human brain with enhanced signal intensity at ultrashort echo times on a clinical platform at 3T and 7T. *Magn. Reson. Med.* 61 1279–1285. 10.1002/mrm.21961 19319893

[B22] Morris G. Anderson G. Dean O. Berk M. Galecki P. Martin-Subero M. (2014). The glutathione system: a new drug target in neuroimmune disorders. *Mol. Neurobiol.* 50 1059–1084. 10.1007/s12035-014-8705-x 24752591

[B23] Öz G. Deelchand D. K. Wijnen J. P. Mlynárik V. Xin L. Mekle R. (2020). Advanced single voxel 1H magnetic resonance spectroscopy techniques in humans: experts’ consensus recommendations. *NMR Biomed.* 1–18. 10.1002/nbm.4236 [Epub ahead of print]. 31922301 PMC7347431

[B24] Prinsen H. de Graaf R. A. Mason G. F. Pelletier D. Juchem C. (2017). Reproducibility measurement of glutathione, GABA, and glutamate: towards in vivo neurochemical profiling of multiple sclerosis with MR spectroscopy at 7T. *J. Magn. Reson. Imaging* 45 187–198. 10.1002/jmri.25356 27351712 PMC5167659

[B25] Provencher S. W. (1993). Estimation of metabolite concentrations from localized in vivo proton NMR spectra. *Magn. Reson. Med.* 30 672–679. 10.1002/mrm.1910300604 8139448

[B26] Rae C. D. Williams S. R. (2017). Glutathione in the human brain: review of its roles and measurement by magnetic resonance spectroscopy. *Anal. Biochem.* 529 127–143. 10.1016/j.ab.2016.12.022 28034792

[B27] Shrout P. E. Fleiss J. L. (1979). Intraclass correlations: uses in assessing rater reliability. *Psychol. Bull.* 86 420–428. 10.1037/0033-2909.86.2.420 18839484

[B28] Smith S. M. (2002). Fast robust automated brain extraction. *Hum. Brain Mapp*. 17 143–155. 10.1002/hbm.10062 12391568 PMC6871816

[B29] Stephenson M. C. (2011). Applications of multi-nuclear magnetic resonance spectroscopy at 7T. *World J. Radiol.* 3:105. 10.4329/wjr.v3.i4.105 21532871 PMC3084434

[B30] Suri S. Emir U. Stagg C. J. Near J. Mekle R. Schubert F. (2017). Effect of age and the APOE gene on metabolite concentrations in the posterior cingulate cortex. *Neuroimage* 152 509–516. 10.1016/j.neuroimage.2017.03.031 28323160 PMC5440729

[B31] Terpstra M. Cheong I. Lyu T. Deelchand D. K. Emir U. E. Bednarik P. (2016). Test-retest reproducibility of neurochemical profiles with short-echo, single voxel MRS at 3T and 7T. *Magn. Reson. Med.* 76 1083–1091. 10.1016/j.physbeh.2017.03.040 26502373 PMC4846596

[B32] Tkáč I. Andersen P. Adriany G. Merkle H. Uğurbil K. Gruetter R. (2001). In vivo 1H NMR spectroscopy of the human brain at 7 T. *Magn. Reson. Med.* 46 451–456. 10.1002/mrm.1213 11550235

[B33] Wijtenburg S. A. Gaston F. E. Spieker E. A. Korenic S. A. Kochunov P. Hong L. E. (2014). Reproducibility of phase rotation STEAM at 3T: focus on glutathione. *Magn. Reson. Med.* 72 603–609. 10.1002/mrm.24959 24151202 PMC3995860

[B34] Wijtenburg S. A. Near J. Korenic S. A. Gaston F. E. Chen H. Mikkelsen M. (2019a). Comparing the reproducibility of commonly used magnetic resonance spectroscopy techniques to quantify cerebral glutathione. *J. Magn. Reson. Imaging* 49 176–183. 10.1002/jmri.26046 29659065 PMC6191387

[B35] Wijtenburg S. A. Rowland L. M. Oeltzschner G. Barker P. B. Workman C. I. Smith G. S. (2019b). Reproducibility of brain MRS in older healthy adults at 7T. *NMR Biomed.* 32 1–8. 10.1002/nbm.4040 30489668 PMC6324949

[B36] Wijtenburg S. A. Rowland L. M. Edden R. A. E. Barker P. B. (2013). Reproducibility of brain spectroscopy at 7T using conventional localization and spectral editing techniques. *J. Magn. Reson. Imaging* 38 460–467. 10.1002/jmri.23997 23292856 PMC3620961

[B37] Zhang Y. Brady M. Smith S. (2001). Segmentation of brain MR images through a hidden Markov random field model and the expectation-maximization algorithm. *IEEE Trans. Med. Imaging* 20 45–57. 10.1109/42.906424 11293691

