# Supplementary material for: Reproducibility of Glutamate, Glutathione, and GABA Measurements in vivo by Single-Voxel STEAM Magnetic Resonance Spectroscopy at 7-Tesla in Healthy Individuals
Source: Front Neurosci. 2020 Sep 15;14:566643. doi: 10.3389/fnins.2020.566643 (PMC7522573; doi:10.3389/fnins.2020.566643)
Supplement: TABLE S1 — Other consistently acquired metabolites. NAA, N-acetylaspartate; GLN, Glutamine; INS, Inositol; Total Cr, Creatine + Phosphocreatine. [file Table_1.pdf]

Supplementary Table 1 – Other consistently acquired metabolites

(NAA = N-acetylaspartate; GLN = Glutamine; INS = Inositol; Total Cr = Creatine + Phosphocreatine)

Variable: Mean(±SD)	NAA	GLN	INS	Total Cr
Concentration - Day 1/Scan 1 (mM)	12.01±0.81	2.27±0.49	7.67±1.20	8.94±0.78
Concentration - Day 1/Scan 2 (mM)	11.46±0.93	2.13±0.49	7.14±0.76	8.54±0.67
Concentration - Day 2/Scan 1 (mM)	11.93±0.72	2.28±0.23	7.69±0.94	8.81±0.63
Concentration - Day 2/Scan 2 (mM)	12.29±1.64	2.19±0.42	7.59±0.90	9.00±1.34
CoV - Day 1 (both scans)	4.98%±3.19%	8.23%±6.32%	5.01%±5.17%	5.32%±3.70%
CoV - Day 2 (both scans)	6.09%±6.27%	11.63%±8.42%	8.08%±6.12%	6.27%±6.03%
CoV - 1st scans (both days)	3.31%±2.27%	11.14%±10.18%	4.33%±3.12%	2.94%±2.27%
CoV - 2nd scans (both days)	5.90%±5.80%	10.96%±8.67%	6.91%±5.80%	7.33%±7.34%

Supplementary Table 2 - Raw uncorrected water-scaled data

(GABA = γ-amino butyric acid; GLUT = Glutamate; GSH = Glutathione; NAA = N-acetylaspartate; GLN = Glutamine; INS = Inositol; Total Cr = Creatine + Phosphocreatine. For creatine-scaling divide by total creatine data columns)

		Day 1		Day 2				Day 1		Day 2	
**Metabolite**	**Participant**	**1st scan**	**2nd scan**	**1st scan**	**2nd scan**	**Metabolite**	**Participant**	**1st scan**	**2nd scan**	**1st scan**	**2nd scan**
	1	1.242	0.74	1.101	1.343		1	9.740	8.653	9.033	8.673
	2	1.093	1.254	1.461	1.326		2	8.174	8.046	8.504	8.581
	3	0.822	0.813	0.913	0.957		3	8.023	7.622	8.630	8.534
	4	1.068	1.282	1.223	1.127		4	9.019	9.070	8.826	8.957
**GABA**	5	0.941	1.122	0.828	0.977	**NAA**	5	8.387	8.563	7.574	7.533
	6	1.061	1.405	1.125	1.221		6	8.216	9.464	8.796	9.244
	7	1.121	1.241	1.295	1.187		7	7.921	8.814	8.317	8.734
	8	1.064	0.966	0.98	1.017		8	8.017	8.022	8.166	8.127
	9	1.239	1.15	1.143	1.074		9	9.020	9.183	8.439	8.476
	10	1.005	1.024	1.01	1.219		10	7.768	7.879	8.158	8.422
	1	7.827	8.035	7.602	7.865		1	1.147	1.041	1.923	1.442
	2	6.773	6.745	7.387	7.091		2	1.851	1.787	1.551	1.281
	3	6.552	6.064	6.983	6.824		3	1.387	1.264	1.657	1.364
	4	7.71	7.289	7.625	7.718		4	2.073	2.190	1.817	1.918
**GLUT**	5	7.536	7.386	6.874	6.582	**GLN**	5	1.546	1.675	1.590	1.254
	6	7.072	8.039	7.213	7.494		6	1.734	1.939	1.579	1.673
	7	8.02	8.122	7.816	7.909		7	1.774	1.499	1.575	1.387
	8	6.845	6.588	6.624	6.707		8	1.709	1.441	1.573	1.810
	9	7.19	7.213	6.876	6.84		9	1.306	1.559	1.435	1.524
	10	6.915	6.679	7.5	7.579		10	1.287	1.369	1.463	1.535
	1	1.123	1.012	1.072	1.069		1	4.853	4.862	5.438	4.999
	2	1.496	1.326	1.317	1.306		2	5.605	5.530	6.004	5.660
	3	1.295	1.404	1.342	1.161		3	5.483	5.189	5.600	5.558
	4	1.391	1.45	1.389	1.26		4	4.879	4.991	5.092	5.067
**GSH**	5	1.372	1.4	1.229	1.303	**INS**	5	5.962	5.604	5.266	5.440
	6	1.324	1.68	1.1	1.382		6	4.303	4.573	4.149	4.310
	7	1.384	1.381	1.285	1.271		7	6.212	5.763	5.999	5.967
	8	1.284	1.16	1.325	1.469		8	5.100	5.001	4.934	4.734
	9	1.129	1.522	1.127	1.169		9	6.040	6.231	5.943	5.487
	10	1.19	1.278	1.554	1.306		10	5.116	5.333	5.992	5.658
	1	6.124	7.165	6.260	5.912						
	2	6.139	6.165	6.311	6.424						
	3	6.317	5.854	6.489	6.276						
	4	6.340	6.329	6.048	6.224						
**Total Cr**	5	6.799	6.707	6.677	6.242						
	6	6.406	6.354	6.183	6.578						
	7	6.671	6.680	6.373	6.311						
	8	5.765	5.818	5.842	5.807						
	9	6.250	6.543	6.020	6.199						
	10	5.749	5.933	6.136	6.305						
